# Analysis of demand and influencing factors for smart senior care among older adults in underdeveloped regions of western China: a case study of Lanzhou

**DOI:** 10.3389/fpubh.2024.1337584

**Published:** 2024-06-12

**Authors:** Yunhua Wang, Hongyu Zeng, Fengli Lv, Jiancheng Wang

**Affiliations:** ^1^School of Management, Lanzhou University, Lanzhou, China; ^2^School of Public Health, Lanzhou University, Lanzhou, China; ^3^Gansu Health Vocational College, Lanzhou, China

**Keywords:** smart senior care, older adults, demand, influencing factors, Lanzhou

## Abstract

**Introduction:**

With the rapid development of artificial intelligence and Internet-of-Things technology, internal support systems among families are gradually weakening, which can no longer satisfy the current demands of older adults. In this context, smart senior care has become a new development direction. However, existing studies on the demand for smart senior care are primarily concentrated in economically developed provinces and mega-cities in eastern China; their research results or conclusions may not apply to underdeveloped areas in the Western region. Therefore, our study selects Lanzhou as a representative city in an underdeveloped western region to investigate the demand of older adults for smart senior care and analyze the influencing factors.

**Methods:**

This cross-sectional study included 4,815 older adults from Lanzhou, China. A structured questionnaire was designed to investigate the demands of the older adults for smart senior care and analyze thie influencing factors. The Chi-square test was used for single factor analysis of each variable. The logistic regression model included the statistically significant variables to analyze factors influencing older adults’ demand for smart senior care. A significance level of *p* < 0.05 was considered statistically significant.

**Results:**

Among the surveyed older adults, 1,625 (33.75%) expressed a demand for smart senior care. The finding indicated that participants’ age, level of education, marital status, monthly income, number of children, type of endowment insurance, and knowledge of smart senior care were significantly associated with their demands for smart senior care (*p* < 0.05). Notably, medical care emerged as the smart senior care service with the highest demand rate (79.45%).

**Conclusion:**

In Lanzhou, older adults show a low level of knowledge but a high demand for smart senior care. Their demand is influenced by personal, family, health conditions, senior care security, and other factors. To advance smart senior care, government departments should accelerate the improvement of the laws and regulations on smart senior care while vigorously enhancing the service’s publicity to raise knowledge about it. Additionally, the service contents for smart senior care should be expanded to meet the diversified demands of older adults.

## Introduction

With the continuous development of the social economy and improvement of medical standards, global life expectancy is steadily increasing, posing significant challenges worldwide due to population aging (1, 2). In June 2019, the United Nations released the “World Population Prospects 2019: Highlights,” indicating a deepening global aging trend, with the 65 and over age group becoming the fastest-growing segment (3). As older individuals require higher levels of medical and long-term care services, the rising proportion of older populations will inevitably lead to increased global health expenditures, thereby impacting the cost of national healthcare systems. The heavy burden of population aging on healthcare support in various countries has become a significant challenge to the sustainability of public finances. This phenomenon is particularly prominent in China, where, over the past 40 years, the country has experienced economic growth and a rapid increase in population aging (4, 5). China currently has the highest older population in the world. From 2000 to 2022, the proportion of older individuals aged 65 and above in China’s total population has surged from 6.96 to 14.86%, marking an increase of nearly eight percentage points (6). China is undergoing the fastest and largest-scale population aging process globally.

With the ever-increasing number of older individuals in China, concerns about senior care and health are becoming more pronounced. On the one hand, the proportion of individuals over 65 is steadily rising, and according to statistics from the National Commission on Aging, as of 2020, China had over 42 million people aged 60 and above who are incapable of self-care, accounting for approximately 16.6% of the older population (7). As individuals age, various aspects of their physical capabilities gradually decline, making them more susceptible to illnesses and disabilities. Moreover, these illnesses are diverse and complex and significantly impact the quality of life in old age. On the other hand, due to the implementation of China’s family planning policy and rapid socioeconomic development, significant changes have occurred in people’s attitudes toward childbirth. China has experienced a trend of smaller family structures, evident in the “4–2-1 family” model, which comprises four older family members, a couple, and one child (8, 9). Simultaneously, as industrialization, urbanization, and modernization advance in China, population mobility has increased significantly. Many working-age individuals have migrated from rural areas to cities and from less developed western regions to more developed eastern regions and mega-cities (10). These combined factors have gradually weakened the traditional family-based senior care system, which can no longer satisfy the current demands of older adults.

As the number of older adults with advanced age, dementia, and disabilities continues to rise in China, there is an urgent need for high-tech, intelligent senior care products and a large number of professional healthcare personnel to provide personalized and comprehensive medical, rehabilitation, and nursing services to the older adults. With the rapid development of big data, the Internet of Things, and artificial intelligence technologies, coupled with the deep integration of traditional senior care services and new technologies, the concept of smart senior care has emerged (11). The smart senior care model is based on advanced technologies such as medicine, the Internet of Things, cloud computing, big data, and mobile internet. It collects, organizes, and identifies dynamic data related to the needs of the older people, offering intelligent, specialized, personalized, and diversified senior care services that encompass daily living support, medical care, leisure and entertainment, and emotional comfort, among other areas (11–13). This aims to meet the growing demands for senior care services. Relevant studies have indicated that the integration of healthy lifestyles with smart technologies has a long-term impact on the aging process of individuals. In this context, telemedicine and the Internet of Things (IoT) technologies applied to health devices are crucial in activating innovative and intelligent living environments. Moreover, policies that utilize new technologies to improve access to healthcare services for the older people can effectively alleviate the heavy burden of population aging on national healthcare expenditures by meeting the healthcare needs of older adults (14). Through the application of information technology, smart senior care can address the needs of older individuals, particularly those with advanced age, dementia, and disabilities, who may lack care and emotional support due to the accelerated population mobility and the trend of smaller family structures. It is one of the practical approaches for China to enhance the governance of an aging society and proactively address population aging. Smart senior care can significantly improve older adults’ quality of life, satisfaction, and happiness.

In recent years, various scholars have conducted extensive research on the demand for smart senior care services and the factors influencing this demand. Huang et al. (11) surveyed 760 older residents in Xuzhou, Jiangsu Province. Their findings revealed that age, the number of children, the frequency of children visiting their parents, self-rated health, the presence of chronic diseases, and attitudes toward smart senior care significantly influence older individuals’ willingness to choose smart senior care services. Sun et al. (1) surveyed 669 older residents in 13 cities in Heilongjiang Province. They found that gender, age, education level, physical health, and living conditions affect the willingness of the older people to use the Internet (1). Luo and Meng (15) conducted research among 204 individuals aged 60 and above in 15 districts of Shanghai. They discovered that age, the number of children, living arrangements, and service costs impact the older adults’ willingness to use smart senior care services. Yep et al. (16) conducted a systematic review of the factors influencing the use of smart senior care products by the older adults. Their findings indicated that age, physical condition, education level, place of residence, gender, cognitive awareness of smart senior care, and self-rated health affect the willingness of the older adults to use smart senior care products. Kong et al. (12) interviewed 15 older individuals living alone in the southwestern region of China. They found that older individuals living alone have higher demands for medical and emergency care, and smart senior care can provide emergency medical assistance, creating a safe and reliable environment for them. Klimova’s research, based on a survey of 224 older individuals in the Czech Republic, demonstrated that income has a significant impact on the acceptance of smart senior care (17). Xiong et al. (18) surveyed 40 respondents and found that women have a significantly higher demand for and acceptance of smart senior care devices. Furthermore, Chen et al.’s (19) study indicated that the acceptance of smart health management technology by the older people is influenced by age, education level, location, and health condition. As individuals age and their mobility decreases while health deteriorates, the demand for smart senior care continues to rise. Bai and Zhu (20) surveyed over 13,000 older users in Jianghan District, Wuhan, analyzing their demand for smart senior care services. The results showed that age, living conditions, and physical health significantly affect the acceptance of smart senior care services among the older adults. Huang et al. (21) investigated the usage intentions of 1,180 older residents in 12 communities in Chongqing regarding smart products. They discovered that factors such as gender, education level, marital status, monthly income, type of health insurance, self-rated health, and the presence of chronic diseases influence the willingness of the older adults to use smart products (21).

In summary, existing research generally acknowledges that factors such as gender, age, number of children, living arrangements, economic affordability, healthcare and senior care security, self-rated health status, the presence of chronic illnesses, and awareness of smart senior care can influence the demand for smart senior care among the older adults. However, current research has some limitations. Specifically, existing surveys on the demand for smart senior care are primarily concentrated in economically developed provinces and mega-cities in eastern China. These provinces or cities have more mature smart senior care models, but their economic development and population characteristics significantly differ from those in less developed western regions. Therefore, their research results or conclusions may not be applicable to underdeveloped areas in the western region. Additionally, the existing studies surveyed a relatively small number of older participants, with most sample sizes being less than 1,000. There is a lack of large-sample surveys specifically targeting the smart senior care demands of older adults, which cannot adequately reflect the demand for smart senior care services among older adults in China.

**Figure 1:**
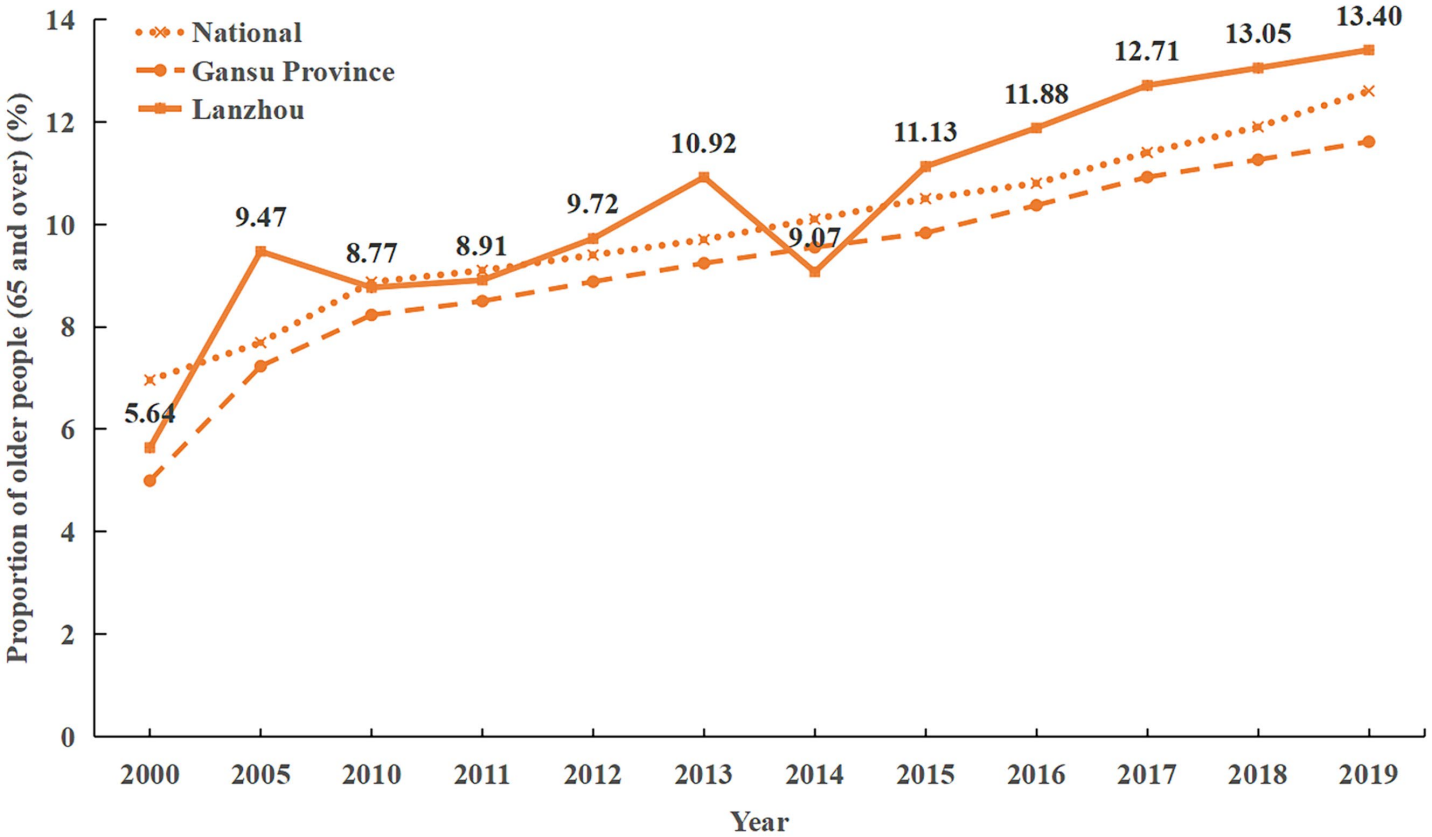
Population growth trends in China, Gansu Province, and Lanzhou, 2000–2019.

Gansu Province, serving as a crucial gateway on the “Silk Road Economic Belt” of “The Belt and Road,” holds a distinctive geographical position and suffers from relative economic underdevelopment. The issue of population aging is particularly acute in this region, categorizing it as a typical case of “aging before prosperity.” According to data from the Seventh National Census, as of the end of 2020, individuals aged 60 and above comprised 17.03% of the total population in Gansu Province, with those aged 65 and above accounting for 12.58% (22). These figures underscore Gansu Province’s transition into an aging society. Lanzhou, the provincial capital of Gansu Province, stands out from other cities due to its large population base and high population density. By the end of 2020, Lanzhou’s population aged 65 and above reached 510,000 individuals, representing 11.70% of the total population. This marks a 2.93-percentage-point increase compared to the Sixth National Census conducted in 2010 (23). Furthermore, an analysis of population aging trends in Lanzhou from 2000 to 2019 reveals a significant surge. In 2000, Lanzhou’s population aged 65 and above stood at 177,200 individuals, accounting for 5.64% of the total population. By 2019, this demographic group had surged to 508,000 individuals, constituting 13.40% of the total population (can be seen in Figure 1). Over two decades (2000–2019), the population aged 65 and above in Lanzhou has tripled, highlighting the pressing nature of population aging in the region. This demographic shift necessitates attention and underscores the urgency of addressing the challenges posed by an aging population. Meanwhile, factors such as low temperatures, economic underdevelopment, and other circumstances contribute to a higher probability of older adults developing chronic conditions such as cardiovascular diseases and diabetes. Additionally, a significant proportion of the population is engaged in migrant labor, and a substantial outflow of young laborers exacerbates the trend of population aging, seniority, morbidity, and empty nesting. Consequently, the phenomenon of “aging before prosperity” becomes increasingly prominent. Against this backdrop, there is a significant gap in the older adults’ needs for daily living, healthcare, and other aspects of senior care (24). Moreover, as individuals age, their risk of partial or complete disability increases. Additionally, the number of children available to provide care has decreased compared to the period before the implementation of the “one-child policy.” This situation results in some older adults not receiving comprehensive care promptly when they fall ill because their children may not be able to accompany them due to their livelihoods. Furthermore, there is a gradual shift in senior care needs toward diversification, high quality, and intelligence, indicating substantial market potential for smart senior care solutions.

In April 2021, Lanzhou City released its “Fourteenth Five-Year Plan and Vision 2035 Outline for National Economic and Social Development,” emphasizing the urgent need for the comprehensive establishment of a family service system and the development of a family service information platform. This platform aims to provide convenient services for older adults. It strongly advocates for promoting smart home-based senior care services to ensure older adults have access to care and support. Currently, Lanzhou is actively responding to the national call by constructing a four-tier senior care information platform spanning across urban, district, street, and community levels. This initiative involves the collaboration of multiple departments, including civil affairs, health, and public security, to establish a comprehensive senior care service information platform. This platform aims to support older adults’ health management, medical services, and medication procurement needs (25). However, despite these efforts, Lanzhou City’s development of smart senior care lags, and the infrastructure remains relatively weak, with numerous challenges yet to be addressed. Therefore, we have chosen Lanzhou, a typical underdeveloped city in western China, as our research site to investigate the need for smart senior care among older adults and analyze the factors influencing this need. By conducting research on the smart senior care needs of the older population and understanding the factors affecting these needs, this study aims to provide empirical data and reference for government policymaking, thereby playing a significant role in promoting the development of smart senior care models and the senior care service industry in China.

## Methods

### Study design and participants

**Figure 2:**
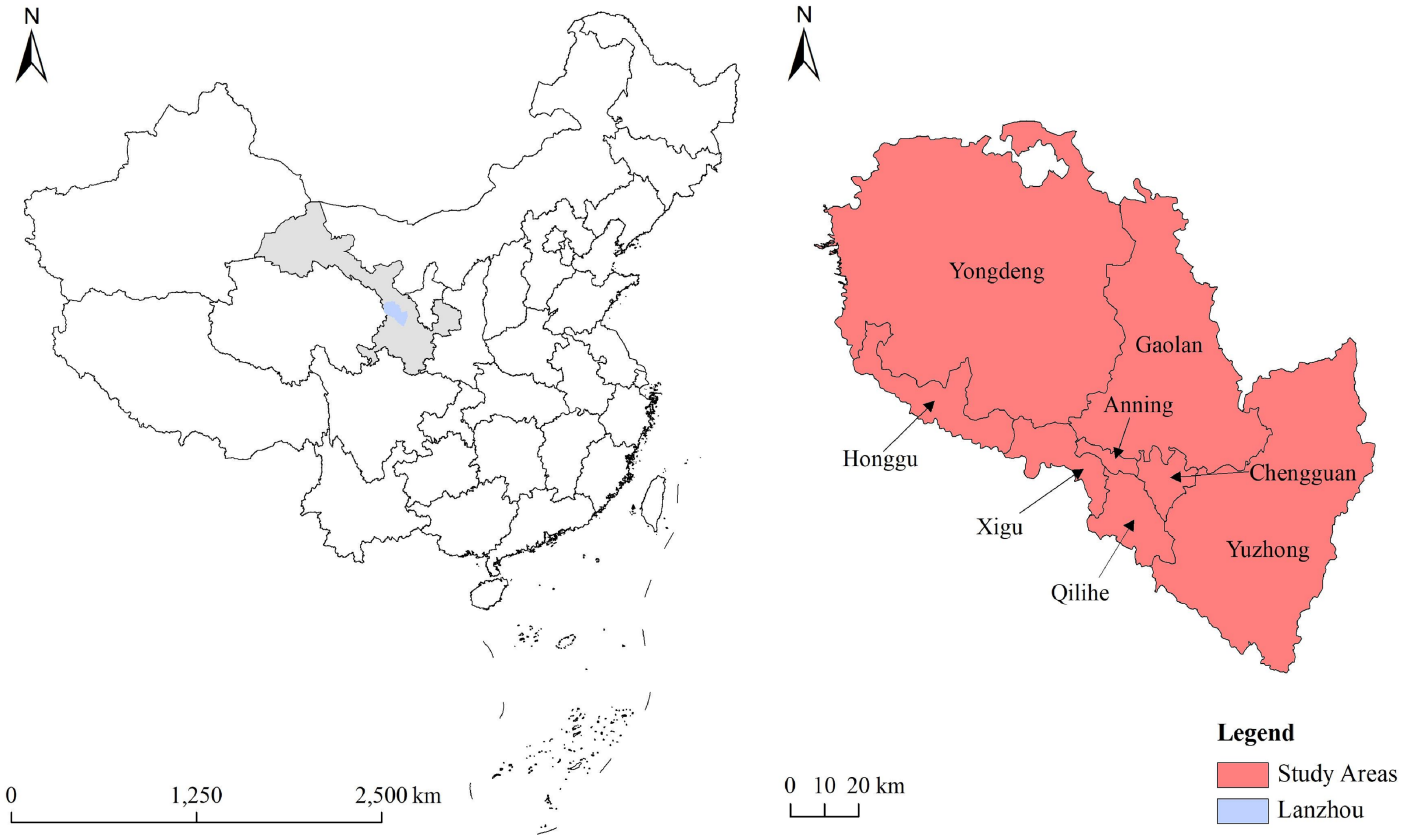
The geographical location of the study area in China.

This study employed a stratified random sampling method to conduct surveys in 8 districts and counties (Chengguan, Qilihe, Xigu, Anning, Honggu, Yongdeng, Yuzhong, and Gaolan) under the jurisdiction of Lanzhou City. The geographical map of the study area is illustrated in Figure 2. While different countries and regions have varying criteria for defining older adults, there are also internationally recognized standards. For instance, the World Health Organization defines individuals aged 60 and above as older adults (26). In China, the criteria and policies regarding older adults differ, and according to the “Law of the People’s Republic of China on the Protection of the Rights and Interests of the Older People,” individuals aged 60 and above are considered older adults (27). Therefore, based on reference to the World Health Organization and relevant Chinese laws, this study defines older adults as individuals aged 60 and above. The inclusion criteria for the study participants are as follows: ① residence in Lanzhou for at least one year, ② absence of visual or auditory impairments, cognitive disorders, or other serious illnesses that may affect the survey, and ③ voluntary participation with informed consent. The survey was conducted from July to August 2020, with 5,000 questionnaires distributed and 4,815 valid questionnaires collected.

### Instruments

To facilitate a comprehensive comparison with existing literature and conduct an in-depth analysis of the demands for smart senior care among older adults in Lanzhou and the influencing factors, the survey instrument utilized in this study was developed collaboratively by epidemiological, statistical experts, and geriatric care management specialists. This instrument was crafted based on a thorough review of relevant literature (1, 2, 8–11, 15, 21, 28–36) and national health policies. The questionnaire items were primarily derived from pertinent literature, the China Health and Retirement Longitudinal Study (CHARLS), and the Fifth Health Services Survey – Family Health Inquiry Questionnaire, as shown in Table 1. In summary, this study used a questionnaire composed of four sections. Section 1 focused on the respondents’ demographic characteristics, included age, gender, level of education, number of children. Section 2 surveyed on the respondents’ socioeconomic status, including marital status, pre-retirement occupation, monthly income, living arrangements. Section 3 assessed the situation of older adults’ health and medical insurance, including self-rated health condition, self-care ability, chronic diseases, type of endowment insurance type of medical insurance. Section 4 assessed the knowledge and demand for the smart senior care among older adults. To assess knowledge about smart senior care, the participants were asked “Do you know about smart senior care?.” The response options ranged from “know very well” to “never heard.” To measure older adults’ demand for the smart senior care, the question of “Do you need smart senior care?” was used. The response was a binary variable (yes or no). For older adults with smart senior care demands, they were asked: “What are your demands for the smart senior care services in the following list?” The list included the medical care, spiritual comfort, leisure and entertainment, emergency ambulance, and life care.

**Table 1:** Summary of questionnaire on the demand and influencing factors for smart senior care among older adults in Lanzhou.

Questionnaire	Number of items	Investigation index	Reference or citing source
Basic demographic characteristics	8	Gender, age, marital status, degree of education, pre-retirement occupation, monthly income, number of children, living arrangement	China Health and Retirement Longitudinal Study (CHARLS) (1, 2, 8–11, 15, 21, 28–36)
Health condition	3	Self-evaluation health condition, incidence of chronic disease, self-care ability	Fifth Health Services Survey – Family Health Inquiry Questionnaire, China Health and Retirement Longitudinal Study (CHARLS) (9, 11, 21, 35)
Medical and endowment insurance	2	Type of medical insurance, type of endowment insurance	Fifth Health Services Survey – Family Health Inquiry Questionnaire, China Health and Retirement Longitudinal Study (CHARLS) (2, 10, 21, 35)
Knowledge and demand for smart senior care	3	Knowledge for smart senior care, demand for smart senior care, service items expected to be provided	(1, 2, 15, 35)

### Quality control

The survey was conducted by trained and evaluated interviewers who underwent standardized training. Ensure that the questionnaire is formulated with concise wording and clear language, facilitating respondents’ comprehension and ensuring completion within a 20-min timeframe. The duration of the interviews was strictly controlled by interviewers who had undergone standardized training. Considering the limited knowledge of older adults regarding smart senior care, interviewers provided guidance and explanations during the questionnaire completion process to ensure comprehensive understanding. After collecting questionnaires, interviewers rigorously screened them based on the completeness and quality of their responses. Criteria for invalid questionnaires included missing answers, highly regular response patterns, or response times exceeding 20 min. Invalid questionnaires were excluded from the data analysis process. After completing the survey for all participants, interviewers collected and inspected the questionnaires.

### Statistical analysis

The data was entered and proofread in duplicate using Epidata 3.1. Subsequently, quality control measures were employed to identify and exclude logical errors and outliers. Following coding, the information was imported into the statistical software SPSS 24.0 for analysis. The enumeration data were analyzed with the *χ*^2^ test, while the influencing factors were examined through binomial logistic regression. A significance level of *p* < 0.05 was considered statistically significant.

## Results

### Sociodemographic characteristics of older adults

#### Basic personal characteristics

In this study, the basic sociodemographic characteristics of older adults investigated can be seen in Table 2. Of the 4,815 older adults surveyed, there were 2,320 males (48.18%) and 2,495 females (51.82%). The number of women was slightly higher than that of men. In terms of age, older adults aged between 60 and 70 years old are 2,703 (56.14%), aged between 71 and 80 years old are 1703 (35.37%), aged between 81 and 90 years old are 387 (8.04%), aged more than 91 years old are the lowest, only 22 (0.45%). It means that most of the respondents of this survey are young older adults. Regarding educational level, 1,929 (40.06%) had education up to elementary school, 1,188 (24.67%) had education up to junior high school, 984 (20.44%) had high school or technical secondary education, 315 (6.54%) had education up to junior college, and 399 (8.29%) had a bachelor’s degree or above. Regarding the number of children, 188 (3.90%) of the surveyed older adults have no children, 1,023 (21.25%) have one child, 1,874 (38.92%) have two children, and 1,730 (35.93%) have three or more children.

**Table 2:** Single factor analysis of the demand for smart senior care among the older adults.

Independent variable	Number	%	Demand rate (%)	Chi-square	*p*
Gender				1.288	0.525
Male	2320	48.18	774 (33.36)		
Female	2495	51.82	851 (34.11)		
Age (years)				101.059	0.000
60 ~ 70	2703	56.14	654 (24.20)		
71 ~ 80	1703	35.37	782 (45.92)		
81 ~ 90	387	8.04	178 (45.99)		
91~	22	0.45	11 (50.00)		
Degree of education				138.991	0.000
Elementary school and below	1929	40.06	287 (14.88)		
Junior high school	1188	24.67	403 (33.92)		
Senior high school or technical secondary school	984	20.44	499 (50.71)		
Junior college	315	6.54	169 (53.65)		
Bachelor degree or above	399	8.29	267 (66.92)		
Marital status				42.012	0.000
Unmarried	16	0.33	5 (31.25)		
Married	3803	78.98	1423 (37.42)		
Widowed	956	19.85	164 (17.15)		
Divorced	40	0.84	33 (82.50)		
Pre-retirement occupation				75.656	0.000
State functionary	194	4.03	118 (60.82)		
Public institution personnel	978	20.31	483 (49.39)		
Enterprise staffs	1056	21.93	497 (47.06)		
Other	2587	53.73	527 (20.37)		
Monthly income (RMB)				96.954	0.000
≤2000	2504	52.01	506 (20.21)		
2001–4,000	1575	32.71	705 (44.76)		
4,001–6,000	553	11.48	292 (52.80)		
≥6,000	183	3.80	122 (66.67)		
Living arrangement				3.081	0.214
Living alone	934	19.40	382 (40.90)		
Not living alone	3881	80.60	1243 (32.03)		
Number of children				115.566	0.000
0	188	3.90	140 (74.47)		
1	1023	21.25	545 (53.27)		
2	1874	38.92	710 (37.89)		
≥3	1730	35.93	230 (13.29)		
Self-rated health				22.600	0.001
Good	1537	31.92	664 (43.20)		
Fair	2173	45.12	720 (33.13)		
Poor	1050	21.81	235 (22.38)		
Very bad	55	1.15	6 (10.91)		
Have chronic diseases				13.803	0.001
Yes	2504	52.00	719 (28.71)		
No	2311	48.00	906 (39.20)		
Self-care ability				2.195	0.700
Have self-care ability	4345	90.24	1500 (34.52)		
Need help from others	453	9.41	120 (26.49)		
No self-care ability	17	0.35	8 (47.06)		
Type of endowment Insurance				59.427	0.000
Endowment insurance for urban workers	1758	36.51	782 (44.48)		
Endowment insurance for urban and rural residents	2427	50.40	677 (27.89)		
Enterprise annuity	122	2.53	94 (77.05)		
Commercial insurance	39	0.80	20 (51.28)		
Not have	469	9.76	52 (11.09)		
Type of Medical insurance				31.457	0.000
Medical insurance for urban workers	1819	37.77	830 (45.63)		
Medical insurance for urban and rural residents	2875	59.70	757 (26.33)		
Commercial insurance	55	1.15	25 (45.45)		
Not have	66	1.38	13 (19.70)		
Knowledge of smart senior care				80.027	0.000
Know very well	33	0.69	22 (66.67)		
Know well	398	8.27	238 (59.80)		
Have gained some understanding	492	10.22	249 (50.61)		
Heard but not understood	923	19.17	309 (33.48)		
Never heard	2969	61.65	807 (27.18)		

#### Socioeconomic status

Regarding the marital status of the surveyed older adults, 3,803 (78.98%) are married, 956 (19.85%) are widowed, 40 (0.84%) are divorced, and only 16 (0.33%) are unmarried. Additionally, 194 (4.03%) were state functionaries before retirement, 978 (20.31%) were public institution personnel, 1,056 (21.93%) were enterprise staff, and 2,587 (53.73%) were engaged in other industries before retirement. In this survey, 2,504 (52.01%) older adults have a monthly income of less than 2000 yuan, 1,575 (32.71%) have a monthly income ranging from 2001 to 4,000 yuan, 553 (11.48%) have a monthly income ranging from 4,001 to 6,000 yuan, and only 183 older adults have a monthly income larger than 6,000 yuan, accounting for 3.80%. Regarding living arrangements, 934 (19.40%) lived alone, while 3,881 (80.60%) lived with others.

#### Health and medical insurance status

Describe the health status of older adults from three characteristics: self-rated health, chronic diseases, and self-care ability. Two thousand and one hundred seventy three older adults perceived their health as fair, accounting for 45.12%. Additionally, 1,537 (31.92%) older adults perceived their health as good, while 1,050 (21.81%) perceived it as poor, and 55 (1.15%) considered their health very bad. 2,504 (52.00%) had chronic diseases, while 2,311 (48.00%) did not. Regarding self-care ability, 4,345 older adults had the ability to take care of themselves, accounting for 90.24%. Additionally, 453 (9.41%) needed help from others in daily life, and 17 (0.35%) could not take care of themselves at all.

Medical security status is described as endowment insurance and medical insurance. Two thousand and four hundred seventy seven older adults have basic endowment insurance for urban and rural residents, accounting for 50.40%. Additionally, 1,758 (36.51%) have basic endowment insurance for urban workers, 122 (2.53%) have enterprise annuity endowment insurance, and only 39 (0.80%) have commercial endowment insurance. Moreover, 469 (9.76%) older adults do not have any endowment insurance. Concerning medical insurance, 2,875 older adults have basic medical insurance for urban and rural residents, accounting for 59.70%. Furthermore, 1,819 (37.77%) have basic medical insurance for urban workers, 55 (1.15%) have commercial medical insurance, and 66 individuals (1.38%) do not have any medical insurance.

#### Knowledge and demand for the smart senior care

**Figure 3:**
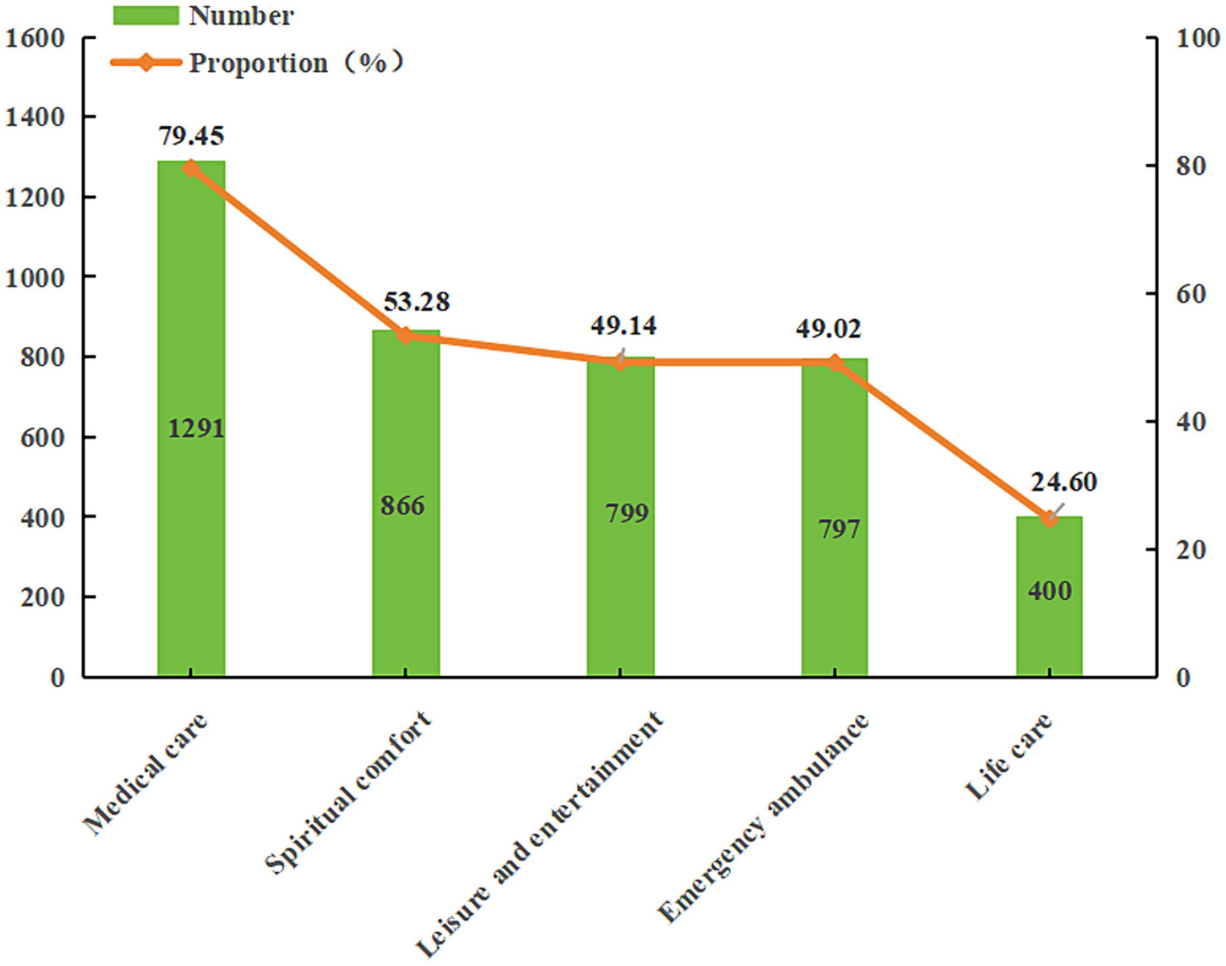
Percentage of participants’ demand for five smart senior care services.

Similarly, Table 2 also shows the knowledge and demand of older adults with different sociodemographic characteristics for the smart senior care. Among the surveyed older adults, 2,969 have never heard of the service model, accounting for 61.65%, 923 (19.17%) older adults have heard but not understood the model, 492 have gained some understanding, accounting for 10.22% and 398 (8.27%) have known it well. However, only 33 (0.69%) older adults reported a comprehensive understanding of this model. Among the surveyed older adults, 1,625 (33.75%) expressed a demand for smart senior care services, while 3,190 (66.25%) indicated no demand. Further investigation of what services are expected by older adults with smart senior care demands. As shown in Figure 3, the older adults in Lanzhou have the highest demand for medical care (79.45%), followed by spiritual comfort (53.28%), leisure and entertainment (49.14%), emergency ambulance (49.02%), and life care (24.60%).

#### Single factor analysis on the demand and influencing factors of the smart older adult care for the older adult in Lanzhou

After the statistical analysis, different ages, education levels, marital status, occupation before retirement, monthly income, number of children, self-rated health, chronic disease, type of endowment insurance, type of medical insurance, and knowledge of smart senior care showed statistical significance (*p* < 0.05) in relation to the needs of older adults for smart senior care in Lanzhou. However, gender, living arrangement, and self-care ability did not exhibit statistical significance (*p* > 0.05). Refer to Table 2 for detailed descriptions.

#### Logistic regression analysis on the demand and influencing factors of the smart senior care for the older adults in Lanzhou

Variables with statistical significance in the single-factor analysis were treated as independent variables (*Xi*), including age, education level, marital status, occupation before retirement, monthly income, number of children, self-rated health, chronic disease, type of endowment insurance, type of medical insurance, knowledge of the smart senior care have statistical significance. The demand of older adults for smart senior care services was designated as the dependent variable *Y* (*Y* = 1 indicating a demand; *Y* = 0 indicating no demand). Subsequently, a binomial logistic regression analysis was conducted. Variable definition for related factors were presented in Table 3.

**Table 3:** Variable definition for the demands and factors for smart senior care among the older adults.

Variable categories	Variable name	Code
Age	X_1_	60 ~ 70 = 1, 71 ~ 80 = 2, 81 ~ 90 = 3, 91 and above = 4
Level of education	X_2_	Bachelor degree or above = 1, Elementary school and below = 2, Junior high school = 3, High school or technical secondary school = 4
Marital status	X_3_	Married = 1, Unmarried = 2, Widowed = 3, Divorced = 4
Pre-retirement occupation	X_4_	State functionary = 1, Public institution personnel = 2, Enterprise staffs = 3, Other = 4
Income per month (RMB)	X_5_	<2000 = 1, 2001 ~ 4,000 = 2, 4,001 ~ 6,000 = 3, >6,000 = 4
Number of children	X_6_	0 = 1, 1 = 2, 2 = 3, ≥3 = 4
Self-rated health	X_7_	Good = 1, Fair = 2, Poor = 3
Have chronic diseases	X_8_	Yes = 1, No = 2
Type of endowment Insurance	X_9_	Not have = 1, Commercial insurance = 2, Enterprise annuity = 3, Endowment insurance for urban and rural residents = 4, Endowment insurance for urban workers = 5
Type of Medical insurance	X_10_	Not have = 1, Commercial insurance = 2, Medical insurance for urban and rural residents = 3, Medical insurance for urban workers = 4
Knowledge of smart senior care	X_11_	Never heard = 1, Heard but not understood = 2, Have gained some understanding = 3, Know well = 4, Know very well = 5
Demand for smart senior care	Y	Yes = 1, No = 0

**Figure 4:**
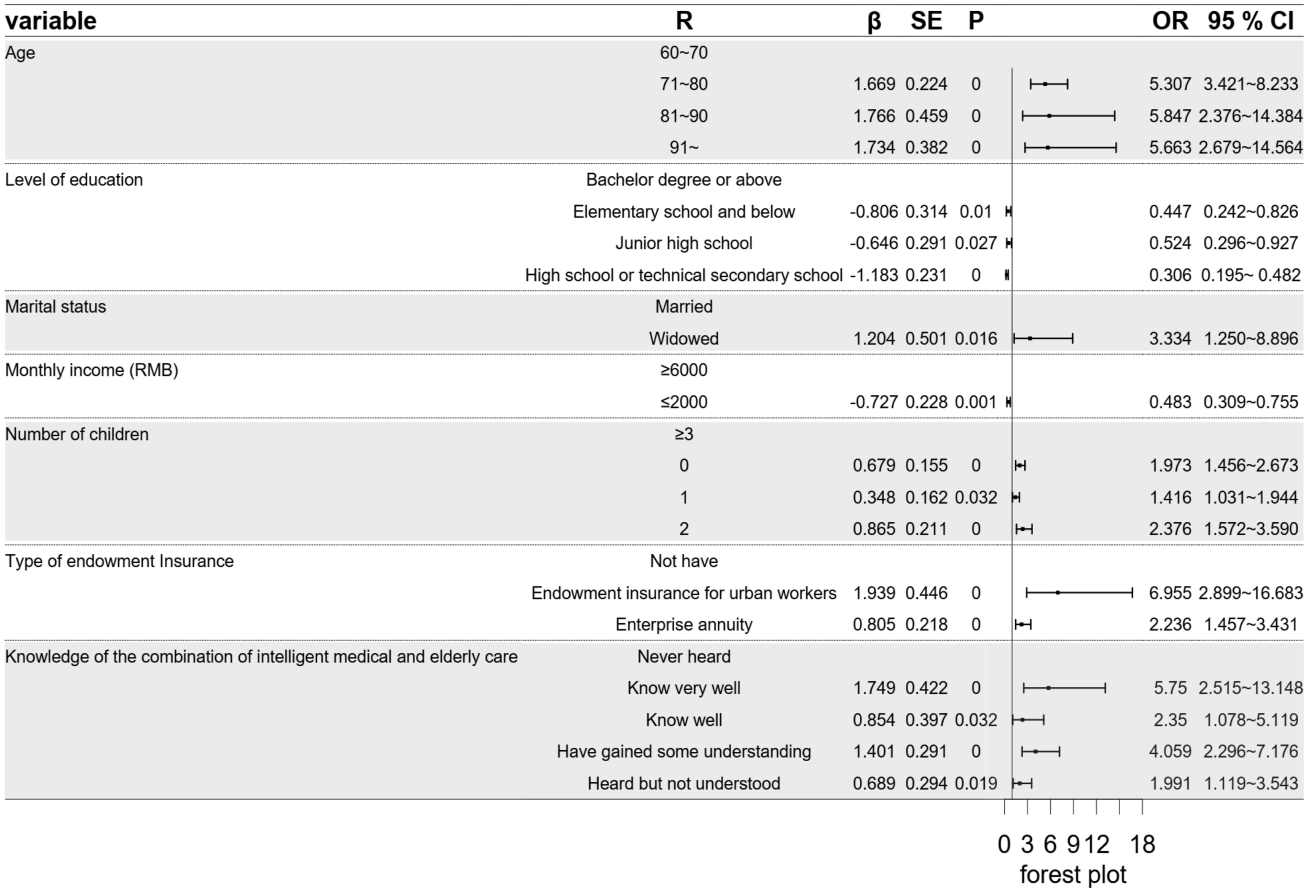
The forest map of influential factors on the demand for smart senior care.

Based on the regression results, older adults in Lanzhou exhibiting certain characteristics are more likely to demand smart senior care. These characteristics include being over 71 years old, widowed, having three children or fewer, having endowment insurance as an urban worker or enterprise annuity, and possessing some understanding of the smart senior care model. Conversely, older adults in Lanzhou with the following characteristics are less likely to demand smart senior care: those with a high school or technical secondary school education or below, and those with a monthly income of 2,000 yuan or below. The regression result is visually presented in Figure 4.

## Discussion

This study investigates the current state and factors influencing the demand for smart senior care among older adults aged ≥60 in Lanzhou. Significant differences were observed in age, level of education, marital status, monthly income, number of children, type of endowment insurance, and knowledge of smart senior care. The findings offer theoretical and practical insights for advancing the smart senior care model in China.

This survey shows that only 33 (0.69%) out of the 4,815 older adults in Lanzhou had a comprehensive understanding of the smart senior care, which is inconsistent with the results of other scholars. Among older residents residents in Xuzhou, 29.9% had just heard of the model of smart senior care, while 6.3% understood the model of smart senior care well (11). Studies conducted by Luo and Meng (15) in Shanghai found that 6.2% of the residents better understood smart senior care services. As observed in the study by Song (28) in Qingdao, 49.17% of older adults knew smart senior care. Wang et al. (29) surveyed 606 older adults in Beijing and found that 3.0% had a relative understanding of smart senior care services, and only 0.8% were very understanding. The study results reveal that older adults in Lanzhou have a lower understanding of smart senior care than in economically developed regions of eastern China. The low knowledge rate of smart senior care among older adults in Lanzhou is caused by many factors. For example, Lanzhou is located in the undeveloped region in western China, with lower economic and social development and educational levels among older adults, resulting in a limited capacity to embrace the new senior care model. In addition, government departments inadequately and ineffectively publicize smart aging policies. Hence, government departments are responsible for promoting smart senior care. Timely release of policies allows older adults quick access to information and effective utilization of the various government-provided benefits. In contrast, older adults receive information through different channels than younger people. Effective strategies involve utilizing television, newspapers, and regular door-to-door campaigns conducted by community or street workers.

This survey indicates that the demand rate for smart senior care among older adults in Lanzhou is 33.75%. Several published studies have also reported the rate of demand for smart senior care among older adults. For example, 90.3% of the older adults have a demand for smart senior care services in Xuzhou (11); 68.7% of the older adults have a demand for gerontechnology in Chongqing (21); 57.7% of the older adults have a demand for smart home care services in Changsha (30); 69.1% of the older adults have a demand for smart senior care in Beijing (29); and 73.38% of the older adults have a demand for smart medical and senior care in Sichuan (31). In other countries, de Veer et al. (37) surveyed 1,014 community-dwelling older adults aged between 57 and 77 in the Netherlands. The study revealed that 63.1% of older adults intended to use e-Health if offered the opportunity. Compared with the above studies, the results of this study show that older adults in Lanzhou have a lower rate of demand for smart senior care. Among the smart senior care services, medical care is in the highest demand and represents a significantly more significant proportion than other service contents. Consistent with previous surveys, medical care services are the most essential service content for older adults. For example, Huang et al. (11) observed that 67.50% of older adults required health care services in Xuzhou. Luo and Meng (15) found that 56.5% of older adults considered medical care services necessary in Shanghai. Yu et al. (31) found that the demand for smart senior care services primarily focused on telemedicine (60.84%), health monitoring (53.99%), and health care (52.85%) in Sichuan. In addition, Majumder et al. (38) conducted a comprehensive review of the latest developments in remote healthcare technologies based on smart homes, emphasizing the significant importance of medical services in smart senior care. The high demand for medical care services among older adults could be attributed to the aging process and physiological decline, leading to poorer health conditions and a higher risk of developing chronic diseases. The Chinese Longitudinal Healthy Longevity Survey (CLHLS) findings indicate that 60.53% of older adults in China are afflicted by chronic diseases (11). The findings of this study also showed that 52.00% of older adults in Lanzhou were suffering from chronic diseases. Therefore, taking advantage of information technology is necessary to ensure the supply of medical care services in smart senior care.

Regarding the basic personal information, age, level of education, and number of children, they have influenced older adults’ demand for smart senior care. Specifically, this study found a higher demand for smart senior care among those over 71 years old, consistent with many studies’ findings (30, 32). Most older adults experience a decline in physical, cognitive, and other functions with age, resulting in a heightened need for smart senior care services, encompassing medical care, assisted living, and other supportive services. Greater demand for higher education was also consistent with previous studies (1, 33). Choi (33) analyzed data from the 2009 US National Health Interview Survey (NHIS) and found that older adults with higher levels of education tend to use smart senior care products more frequently. Dogruel et al. (34) surveyed older media users in Germany and the United States. They found that as the overall level of education among older adults increases and modern multimedia technologies increasingly permeate their daily entertainment, there is a significant rise in demand for intelligent services and products among the older adults (34). Older adults with higher education tend to adapt more quickly to new things, exhibiting a higher demand for smart senior care (30). In addition, this study shows that the number of children influences the demand rate of smart senior care. Older adults with fewer children have an increasing demand for the service. A study by Dermody et al. (13) from Sunshine Coast University in Australia surveyed 19 community-dwelling older adults, revealing that familial support influences seniors’ inclination to opt for smart senior care. It was found that seniors with lesser familial support are more predisposed to embrace smart senior care services (13). This trend might be attributed to the reduction in family size and the rise of empty-nest families (35). The fewer the number of children, the more the older adults lack the companionship and care of their children (30). Under such circumstances, older adults cannot be adequately cared for. Worse still, family members often find it challenging to meet the medical and nursing care needs of older adults, particularly those with chronic illnesses, disabilities, and incapacities. Therefore, older adults with fewer children have a higher demand for smart senior care.

Regarding socioeconomic factors, marital status, and monthly income also influence the demand for smart senior care among older adults. This study shows that widowed older adults have a higher demand for smart senior care than married older adults. Previous studies have supported this conclusion (32). The reason may be that widowed older persons lack care support from their spouses and need to take care of themselves. In addition, widowed older adults are more prone to loneliness and symptoms of psychological disorders. As a result, they are in greater demand of spiritual comfort and leisure and entertainment services than other older adults. Income status is crucial in assessing the affordability of smart senior care services for older adults. This study found that the higher the monthly income, the higher the demand for smart senior care services, consistent with many previous studies’ findings (1). Research indicates that older adults are susceptible to the prices of senior care products and services and are generally unlikely to proactively utilize technologies and services with higher costs (39). A lack of sufficient financial support directly affected purchasing power, restricting the use of smart senior care services. A study by Luo and Meng (15) found that high prices hinder the promotion of smart senior care services. Thus, the relevant government departments should customize affordable services to accommodate the diverse demands of older adults, considering their various ages, living habits, and cognitive abilities.

In this study, the type of endowment insurance is also an essential factor affecting the demand of older adults for smart senior care in Lanzhou. Older adults with insurance for urban workers’ endowment insurance and enterprise annuities have a greater demand for smart senior care. Generally, older adults with urban workers’ endowment insurance and enterprise annuities had formal jobs before retirement, making them more financially able to afford smart senior care services. As a result, older adults with urban workers’ endowment insurance and enterprise annuities exhibit a higher demand for smart senior services. Several studies indicate that medical and endowment insurance influences older adults’ demand for medical and care services (35, 36). Additionally, government departments should enhance the long-term care insurance system, increase the resources for long-term care services, promote long-term care insurance and health sciences, and address the expenses related to primary living care and medical services for older adults (40).

## Limitations

This study has some limitations. Firstly, the respondents of this study came from Lanzhou, where the demographic characteristics of older adults may differ significantly from those in other regions across China in terms of economic, cultural, and social backgrounds. As a typical city in economically underdeveloped western regions, Lanzhou exhibits distinct characteristics among its older adults compared to the more developed eastern provinces and mega-cities. Consequently, the applicability of the study findings on a nationwide scale is restricted. To gain a more comprehensive understanding of the smart senior care needs of Chinese older adults, it is imperative to account for regional disparities and conduct surveys encompassing a broader spectrum of regions, including the eastern, central, and western areas, as well as urban and rural settings. Secondly, while the study attempted to incorporate various factors influencing the smart senior care needs of older adults, such as age, economic status, and health conditions, the analysis lacked a comprehensive approach. Many factors, including individual characteristics, family backgrounds, geographic factors, economic circumstances, and societal influences, influence smart senior care needs. Comprehensively analyze these factors to ensure a more comprehensive understanding of these needs, potentially affecting the targetedness of policy formulation. Lastly, the study employed a cross-sectional survey design. While cross-sectional surveys provide rapid data collection at a specific time, they lack longitudinal tracking of changes in smart senior care needs over time. Given the continuous development of society, economy, and technology, older adults’ needs are subject to change, particularly with rapid advancements in smart technology. Therefore, longitudinal tracking studies are crucial for a comprehensive understanding of the evolving nature of smart senior care needs. They are instrumental in informing the development of forward-looking and targeted policy measures. In conclusion, despite offering insights into the smart senior care needs of older adults in Lanzhou, this study is constrained by the scope of survey subjects, depth of factor analysis, and research methodology. Therefore, this study has some limitations. To achieve a more thorough and accurate understanding of the smart senior care needs of Chinese older adults, improvements and enhancements are required in the selection of survey subjects, comprehensive factor analysis, and research methodologies.

## Conclusion

This study selected Lanzhou, a representative city in the underdeveloped western region of China, as the survey location to investigate the demand for smart senior care among older adults and analyze the influencing factors. The research findings provide data support and a reference framework for government departments to formulate relevant policies. The results indicate that older adults in Lanzhou exhibit a low level of knowledge regarding smart senior care but demonstrate a high demand for such services. Moreover, among the complex factors influencing the demand for smart senior care among older adults, the number of children, age, level of education, monthly income, marital status, type of endowment insurance, and knowledge regarding smart senior care are identified as primary influencing factors. In the future, relevant government departments in Lanzhou should focus on three aspects to meet the demand for smart senior care services among older adults and enhance their overall quality of life in their later years.

Firstly, relevant government departments should strengthen policy guidance and financial subsidies. The development of smart senior care services relies on government policy guidance. On the one hand, government departments should enact laws and regulations explicitly targeting smart senior care, improve the information standard system in the senior care industry, and create a standardized and orderly development environment for smart senior care. Additionally, a sound supervision and evaluation mechanism for senior care services should be established. The government should take on a supervisory role in providing senior care services, strengthening pre-, during-, and post-supervision and evaluation to ensure the quality of smart senior care services. On the other hand, relevant government departments should increase financial investment and enhance financial subsidies. Service pricing is one of the factors influencing the demand for smart senior care services among older adults. If service prices exceed the affordability of older adults, the services will lose their attractiveness. Therefore, special financial funds should be allocated, continuously enhancing financial support for smart senior care services, expanding the scope of financial subsidies, increasing the level of service subsidies, reasonably dividing subsidy levels, expanding the beneficiary groups, enabling more older adults to enjoy government subsidies, and thereby increasing the enthusiasm of older adults to use smart senior care services.

Secondly, innovative means of promotion should be utilized to increase the publicity of smart senior care models. Elevating the cognitive level of older adults regarding smart senior care is crucial since they are the target beneficiaries of such services. On the one hand, efforts should be made to enhance the promotion of smart senior care services through innovative means. This can include offline public lectures, newspapers, community bulletin boards, television, short videos, and other methods to introduce the concept and specific implementation of smart senior care models to older adults. Furthermore, the latest domestic practices should be collected and investigated, with in-depth reporting and interpretation of typical cases that have achieved positive results, gradually deepening the understanding and acceptance of smart senior care models among older adults, thereby increasing their receptiveness. On the other hand, emphasis should be placed on cultivating the internet mindset of older adults. Tailored internet knowledge and skills training should be conducted to enhance the internet literacy and proficiency of older adults, enabling them to understand better and accept innovative senior care services.

Finally, it is essential to accurately grasp the needs of older adults and enrich the content of smart senior care services. Older adults exhibit heterogeneity. Thus, the provision of smart senior care services needs to be tailored to match their personalized and diversified care needs. Providers of smart senior care services should enrich the content based on the actual needs of older adults and strive to enhance the quality of services. This ensures older adults can better enjoy various care needs such as daily assistance, healthcare, and emotional support. In practice, different criteria can be used to classify service recipients. By categorizing older adults according to different consumption levels, age groups, educational levels, and physical health conditions, service providers can tailor their offerings to better meet each group’s needs. Services such as emotional support and recreational activities can be prioritized for economically affluent older adults. For older adults with health issues, medical care and rehabilitation should be emphasized while paying attention to their psychological well-being and providing appropriate emotional support.

## Data availability statement

The raw data supporting the conclusions of this article will be made available by the authors, without undue reservation.

## Ethics statement

The studies involving humans were approved by Gansu Health Vocational College. The studies were conducted in accordance with the local legislation and institutional requirements. The participants provided their written informed consent to participate in this study.

## Author contributions

YW: Conceptualization, Data curation, Writing – original draft. HZ: Data curation, Formal analysis, Investigation, Supervision, Writing – review & editing. FL: Data curation, Formal analysis, Software, Writing – review & editing. JW: Conceptualization, Funding acquisition, Supervision, Writing – original draft.
